# Bacterial Foraging Optimization Based on Self-Adaptive Chemotaxis Strategy

**DOI:** 10.1155/2020/2630104

**Published:** 2020-05-27

**Authors:** Huang Chen, Lide Wang, Jun Di, Shen Ping

**Affiliations:** School of Electrical Engineering, Beijing Jiaotong University, Beijing 100044, China

## Abstract

Bacterial foraging optimization (BFO) algorithm is a novel swarm intelligence optimization algorithm that has been adopted in a wide range of applications. However, at present, the classical BFO algorithm still has two major drawbacks: one is the fixed step size that makes it difficult to balance exploration and exploitation abilities; the other is the weak connection among the bacteria that takes the risk of getting to the local optimum instead of the global optimum. To overcome these two drawbacks of the classical BFO, the BFO based on self-adaptive chemotaxis strategy (SCBFO) is proposed in this paper. In the SCBFO algorithm, the self-adaptive chemotaxis strategy is designed considering two aspects: the self-adaptive swimming based on bacterial search state features and the improvement of chemotaxis flipping based on information exchange strategy. The optimization results of the SCBFO algorithm are analyzed with the CEC 2015 benchmark test set and compared with the results of the classical and other improved BFO algorithms. Through the test and comparison, the SCBFO algorithm proves to be effective in reducing the risk of local convergence, balancing the exploration and the exploitation, and enhancing the stability of the algorithm. Hence, the major contribution in this research is the SCBFO algorithm that provides a novel and practical strategy to deal with more complex optimization tasks.

## 1. Introduction

Bacterial foraging optimization (BFO) algorithm is a novel swarm intelligence optimization algorithm based on the foraging behavior of *E. Coli*, which was proposed by Professor Passino in 2002 [1, 2]. Compared with other optimization algorithms, the BFO algorithm aces in fast convergence and global search by its simple bacterial individual structure and behavior, varied group types and characteristics, and efficient life cycle [3–5] even though it is still in a preliminary stage of research. Therefore, the BFO algorithm has been successfully applied in many fields. Literature in [6–8] adopted the BFO algorithm to optimize the probabilistic planning, load dispatch, reconstruction, and loss minimization of the power energy network. Artificial intelligence learning and robot automatic control can also refer to the BFO algorithm [9–11]. Research in [12, 13] applied the BFO algorithm to the optimization of the wireless network, including the structure design and routing topology.

At present, the research of BFO focuses on improving its performance for more applications. One of the commonly used methods is to adjust the algorithmic logic. For example, Tang et al. [14] proposed an improved multilevel thresholding approach to improve the global search ability of the classical BFO. By adjusting the search scope and chemotaxis variables dynamically, the bacterial population was guided to move towards the global optimum [15]. Besides, the combination of BFO and other algorithms is also an appealing topic. The convergence speed and the local search ability of the BFO algorithm were proved by referring to some other algorithms, such as the neural network in [16], the genetic algorithm (GA) in [17], the particle swarm optimization (PSO), and unit step function in [18], as well as new chemotaxis with the differential evolution (DE) operator in [19, 20].

According to the research on the improvement of the BFO, the classical BFO algorithm still has some drawbacks, in which the fixed chemotactic step size and the weak connection among bacteria matter the most. The first drawback, the fixed chemotactic step size, makes the balance between exploration and exploitation difficult to realize. The second drawback, the weak connection among bacteria, leads to poor randomicity in chemotaxis. When searching in a complex multimodal solution set, the above two drawbacks will lead the bacteria community to the local convergence rather than the global one.

To overcome these drawbacks, this paper proposed the BFO with self-adaptive chemotaxis strategy (SCBFO) as one of the novel BFO algorithms, which improves the classical algorithm theoretically via the following two aspects.

First, a self-adaptive swimming method based on bacterial search state features is proposed to overcome the classical drawback caused by a fixed step size. Three important features of the bacterial search state, the population diversity, the iteration, and the mean fitness are extracted and calculated. They are taken as the inputs of a multidimension fuzzy logic controller (MFLC) to obtain the chemotaxis swimming step size suitable for the current search state.

Then, the chemotaxis flipping is improved in the SCBFO based on information exchange strategy. By introducing the strategy of information exchange between bacteria, the state perception between bacteria is improved so that bacteria can understand the overall optimization state in the optimal solution space and instantly adjust the flipping of bacterial chemotaxis.

Therefore, the SCBFO algorithm can effectively solve the performance degradation caused by the drawbacks of the classical BFO and improve the search performance stability.

This paper is structured as follows: Section 2 analyses the general solving process of the BFO algorithm.  Section 3 proposes the SCBFO algorithm, which is based on the self-adaptive chemotaxis strategy. In Section 4, the SCBFO algorithm is tested using the CEC 2015 benchmark test set and compared with other algorithms, in which the optimization results, the convergence trend, and the performance stability of the algorithm are analyzed.  Section 5 summarizes the performance of the SCBFO algorithm.

## 2. Fundamental Structure of the BFO Algorithm

**Figure 1:**
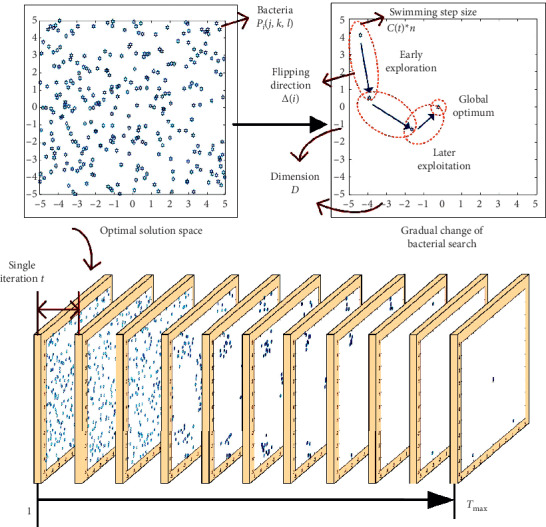
The fundamental structure of the BFO algorithm.

The fundamental structure of the BFO algorithm was proposed in the classical BFO algorithm and is shared by all the improved versions of the BFO algorithm [2, 18]. Thus, the fundamental structure of the BFO algorithm is established in this section alongside essential functions that will be used and optimized in this paper, as shown in Figure 1.

To start with, the initialization of the classical BFO algorithm includes two important contents:

1. Solution space initialization: the solving spatial dimension *D*, range, and mapping function *f*(*x*) are designed.
2. Bacterial initialization: the number of bacteria is nominated by *S.* The position of the *i*th bacterium in the optimization space is expressed as *P*_*i*_ (*j*, *k*, *l*), which equals to the optimal parameter of the solution, i.e., *P*_*i*_ (*j*, *k*, *l*) = [*m*_1_, *m*_2_,…, *m*_*D*_].

Therefore, the fitness of the *i*th bacterium in the optimization space is expressed as *J*_*i*_ (*j*, *k*, *l*), which is determined by the function of the bacterium position in the following:

(1) $$\begin{array}{c} J_{i}\left(j,k,l\right)=f\left(P_{i}\left(j,k,l\right)\right)=f_{i,j,k,l}\left(m_{1},m_{2},\ldots ,m_{D}\right). \end{array}$$

In (1), the lower values of the function indicate the higher fitness [1]. *i* represents the *i*th bacterium, while *j*, *k*, and *l* correspond to the main processes of the BFO algorithm: chemotaxis, reproduction, and elimination and dispersal.

### 2.1. Chemotaxis

The chemotaxis process consists of a great amount of swimming and flipping motions. In the *j*th chemotaxis process, the movement of the *i*th bacterium can be expressed in the following:

(2) $$\begin{array}{c} P_{i}\left(j+1,k,l\right)=P_{i}\left(j,k,l\right)+\frac{\Delta \left(i\right)}{\sqrt{\Delta^{T}\left(i\right)\Delta \left(i\right)}}C\left(i\right)n, \end{array}$$

where the swimming step length of the *i*th bacterium is divided into single swimming step size *C*(*i*) and the number of swimming *n* and Δ(*i*) is the direction vector of the *i*th bacterium in the *p*-dimension optimization space. Each element of Δ(*i*) is a numeric value at the range of [−1,1], whose initialization is set as a random value within the range. When the *i*th bacterium finds a higher fitness position to be a favorable environment during the *j*th chemotaxis, it continues to move in the same direction based on this time. Instead, Δ(*i*) chooses a new random direction.

### 2.2. Swarming

The swarming behavior of the bacteria can be characterized by attraction and repulsion. The numerical relationship can be defined in the following:

(3) $$\begin{array}{c} J_{cc}\left(P_{i}\right)=\sum_{i=1}^{s}\left[-d_{\text{att}}\exp\left(-\omega_{\text{att}}\sum_{m=1}^{p}{\left(P_{i,m}-\bar{P_{m}}\right)}^{2}\right)\right]+\sum_{i=1}^{s}\left[h_{\text{rep}}\exp\left(-\omega_{\text{rep}}\sum_{m=1}^{p}{\left(P_{i.m}-\bar{P_{m}}\right)}^{2}\right)\right], \end{array}$$

where *d*_att_ indicates the depth at which the attracted material is released by the *i*th bacterium, while *ω*_att_ indicates the width of the same attracted material. Similarly, because two bacteria cannot be in exactly the same position, the repulsion is adopted as *h*_rep_ and *ω*_rep_. After the swarming process, the fitness of the *i*th bacterium is shown in the following:

(4) $$\begin{array}{c} J_{i}\left(j+1,k,l\right)=J_{i}\left(j,k,l\right)+J_{cc}\left(P_{i}\left(j,k,l\right)\right). \end{array}$$

### 2.3. Reproduction

The bacteria replicate when they reach a better environment; otherwise, they will pass away. Thus, after the chemotaxis and the swarming process, the fitness of all the bacteria is calculated and sorted. The fitness of the *i*th bacterium is expressed in the following:

(5) $$\begin{array}{c} J_{i,\text{health}}=\sum_{j=1}^{Nc}J_{i}\left(j,k,l\right). \end{array}$$

Half of the bacteria at better condition *S*_*r*_=(*S*/2) are selected to survive, while the other half pass away. The survived bacteria then reproduce into two colonies located in the same region, keeping the total number of bacteria *S* fixed.

### 2.4. Elimination and Dispersal

After the reproduction, each bacterium is dispersed with the probability of *P*_ed_, but the total number of bacteria remains the same. Once a bacterium is eliminated, it will be randomly dispersed to a new location.

(6) $$\begin{array}{c} r=\text{random}\left[0,1\right];\\ P_{i}\left(j,k,l\right)=\left\{\begin{array}{cc} P_{i}\left(j,k,l\right), & r>P_{\text{ed}},\\ \left(m_{1}^{\prime },m_{2}^{\prime },\ldots ,m_{p}^{\prime }\right), & r<P_{\text{ed}}. \end{array}\right. \end{array}$$

As shown in (6), elimination occurs when *r*_*i*_ < *P*_ed_. The original position of the *i*th bacterium *P*_*i*_ was replaced by a new one *P*_*i*_′=(*m*_1_′, *m*_2_′,…, *m*_*D*_′). As a result, the optimal parameter *m* is updated to a random parameter *m*′ that will be solved in the optimization space.

## 3. Improvement Based on Self-Adaptive Chemotaxis Strategy

The chemotaxis process has a great influence on the exploration and exploitation of the BFO algorithm, and it is the most important computing process of the BFO algorithm in searching the optimization space. The effect of chemotaxis is mainly achieved by two operations: swimming step size and flipping direction.

Therefore, to improve the performance of the BFO algorithm, two improvements are proposed in this paper, in which the features in the search state of the BFO algorithm are extracted and calculated and the information exchange between bacteria is increased. With these two improvements, the dynamic self-adaptive ability for bacterial swimming and flipping motions is designed, and a novel BFO algorithm, the SCBFO algorithm, is proposed.

### 3.1. Self-Adaptive Swimming Based on Bacterial Search State Features

According to the above research, the single swimming step size *C*(*t*) and the number of swimming *n* of chemotaxis determine the swimming step size of the algorithm. Then, the swimming step size determines whether the search performance of the algorithm can adapt to the current search state. When the search state is in the early stage, the algorithm needs the exploration ability for global search; then, in the later stage, the exploitation ability is required for local development.

For different optimization problems, the change of the BFO search state is also different. Meanwhile, because the chemotaxis process is nonlinear, it is so complex that the transition from the global exploration to the local exploitation cannot be simply described and divided by the means of analytical equations.

**Figure 2:**
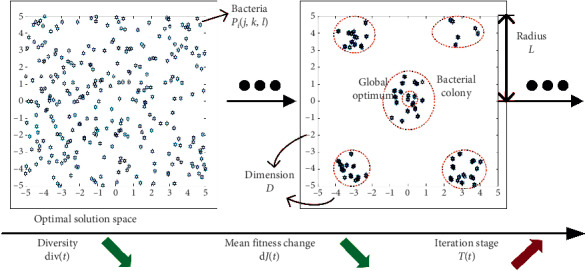
The bacterial population status of the BFO algorithm.

Therefore, to realize the dynamic adjustment of the BFO algorithm to the appropriate chemotaxis swimming, this paper extracts three important features of the BFO in each search state, including population diversity, iteration, and mean fitness, as shown in Figure 2.

The population diversity of bacteria describes the dispersion of the bacteria. The population diversity will get up to a higher level if the bacteria disperse at a wider range, and vice versa. This paper measures the population diversity of the bacterial colony in the chemotaxis process of BFO as

(7) $$\begin{array}{c} \text{div}\left(t\right)=\frac{1}{D\times S}g\sqrt{{\sum_{i=1}^{S}\left(\frac{P_{{}^{i}}\left(j,k,l\right)-\bar{P_{{}^{i}}\left(j,k,l\right)}}{\left|L\right|}\right)}^{2}}, \end{array}$$

where *L* represents the longest radius in the solution space and div(*t*) within the range of [0,1] measures the distance from each bacterium to the center of the population, which is irrelevant to the size of the solution space or the number of bacteria.

The iteration of the BFO algorithm is expressed by a parameter *T*, which is defined as an expression in the range of (0,1] in (8), where *t* and *T*_max_ represent the index of the current chemotaxis and the maximum iteration, respectively. Thus, the definition of parameter *T* is generally suitable to different algorithms no matter how the parameters, the dimension, and the solution space are set in the algorithms:

(8) $$\begin{array}{c} T\left(t\right)=\frac{t}{T_{\max}}. \end{array}$$

The change of the mean fitness in two chemotaxis processes, d*J*, is mainly investigated as one of the crucial standards to evaluate the BFO algorithm [21]. To give a general definition, the change of the mean fitness d*J* is defined in the per-unit form within [−1,1] seen in the following:

(9) $$\begin{array}{c} \text{d}J\left(t\right)=\frac{J\left(t\right)-J\left(t-1\right)}{J_{\max}-J_{\min}}, \end{array}$$

where *J*_max_ and *J*_min_ show the maximum and minimum of the fitness, respectively.

Therefore, in this paper, the three variables, the population diversity in (7), iterations in (8), and the mean fitness of bacteria in (9), are set as inputs of the multidimension fuzzy logic controller (MFLC), which is designed to investigate the search status of the algorithm in this paper. Then, with the two outputs of the MFLC, the chemotaxis swimming processes are adjusted in the following:

(10) $$\begin{array}{c} n\left(t+1\right)=n\left(t\right)+\text{d}n\left(t\right),\\ C\left(t+1\right)=C\left(t\right)gC_{\text{Multi}}\left(t\right), \end{array}$$

where the two output variables of the controller are the bacterial swimming activity increment d*n*(*t*) ∈ [−0.01, 0.01] and the bacterial swimming step multiple *C*_Multi_(*t*) ∈ (0,1].

The variable sets of the MFLC are shown in Table 1.

The fuzzy rules of the variables may be expressed in Table 2.

**Figure 3:**
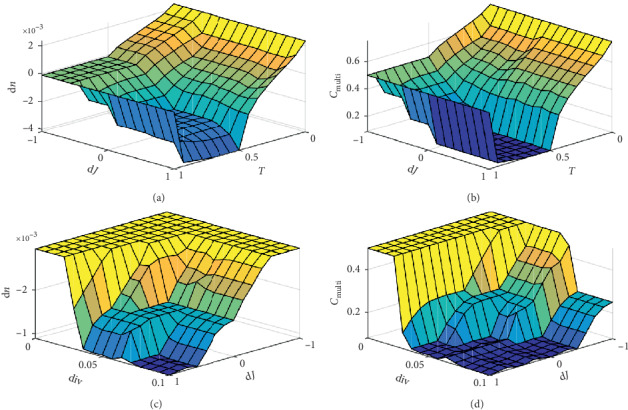
Membership status of MFLC.

According to the aforementioned principles, the membership function of each variable can be combined to form a complete function corresponding to the inputs and outputs. As is shown in Figure 3, under the control of MFLC, the single swimming step size *C*(*t*) and the number of swimming *n* can be dynamically adjusted in different stages.

### 3.2. Improvement of Chemotaxis Flipping Based on Information Exchange Strategy

The flipping of the bacteria is another important operation during the chemotaxis process of the BFO algorithm. Each bacterium controls its own chemotaxis direction based on the extremum found during its swimming. The flipping variable is Δ(*i*) in (2). Although this method is beneficial to the randomness of the search, the blocked information among the bacteria slows down the searching process. Thus, the BFO algorithm with (2) suffers the disadvantage of falling into the local optimum.

**Figure 4:**
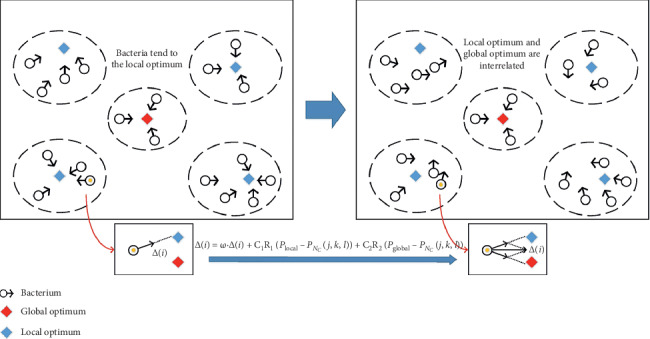
The direction of the SCBFO algorithm.

In order to solve this problem, referring to the information exchanging strategy of individuals in the particle swarm optimization (PSO) algorithm [22, 23], the flipping variable Δ(*i*) in the BFO algorithm is updated in (11) and shown in Figure 4:

(11) $$\begin{array}{c} \Delta_{t+1}\left(i\right)=\omega g\Delta_{t}\left(i\right)+C_{1}R_{1}\left(P_{\text{local}}-P_{N_{c}}\right)+C_{2}R_{2}\left(P_{\text{global}}-P_{N_{c}}\right). \end{array}$$

Coefficients that adjust the process of chemotaxis are utilized in (11): *ω* is the inertia factor, which represents the chemotaxis inertia of the bacteria at a certain direction. *C*_1_ and *C*_2_ are acceleration constants. *C*_1_ represents the rate at which the *i*th bacterium moves towards its individual optimal value *P*_local_ during the process of individual bacterial chemotaxis, while *C*_2_ indicates the adjusting rate to the global optimum value *P*_global_ for all the bacterial chemotaxis. *R*_1_ and *R*_2_ are random values at the range of (0,1), which are used to improve the randomness of the bacterial flipping and enhance the searching ability.

### 3.3. Description of the SCBFO Algorithm

**Figure 5:**
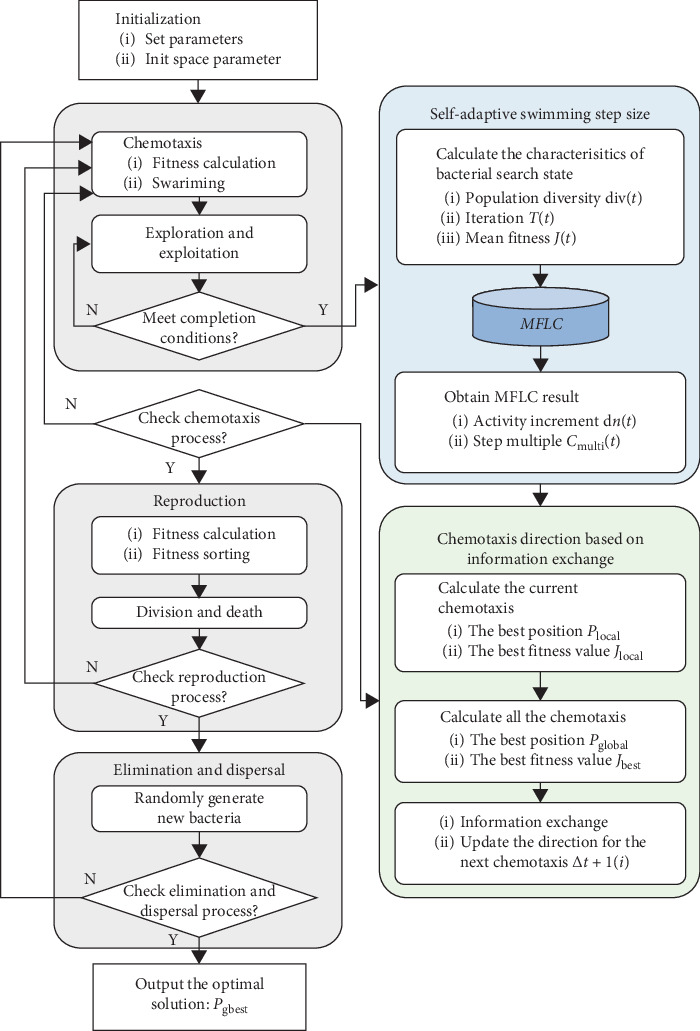
The description of the SCBFO algorithm.

Therefore, based on Sections 3.1 and 3.2, with (7)–(11) and MFLC, the SCBFO algorithm is established via the improvements of the BFO algorithm. The flowchart of the proposed SCBFO algorithm is summarized in Figure 5.

The special steps of the SCBFO algorithm are described as follows:

1. According to formulas (1)–(11), the initial parameters are described in Table 3.
2. The completion conditions of exploration and exploitation steps in the chemotaxis process are as follows:
3. According to formula (2), when the fitness of the *i*th bacterium is worse than the original position after the chemotaxis process or when its number of swimming *n* = 0, the exploration and exploitation steps are terminated, and the next process is started; otherwise, continue.
4. The completion conditions of chemotaxis, reproduction, and elimination and dispersal process are as follows:
5. According to the definition and initialization of bacteria in Section 2, the position of the *i*th bacterium in the optimization space is expressed as *P*_*i*_(*j*, *k*, *l*), *j* represents the *j*th chemotaxis process, *k* represents the *k*th reproduction process, and *l* represents the *l*th elimination and dispersal process. When the count value of the corresponding process is 0, the process is completed, and the next process is started.

## 4. Experiments and Analysis

### 4.1. Test Set and Parameters

**Figure 6:**
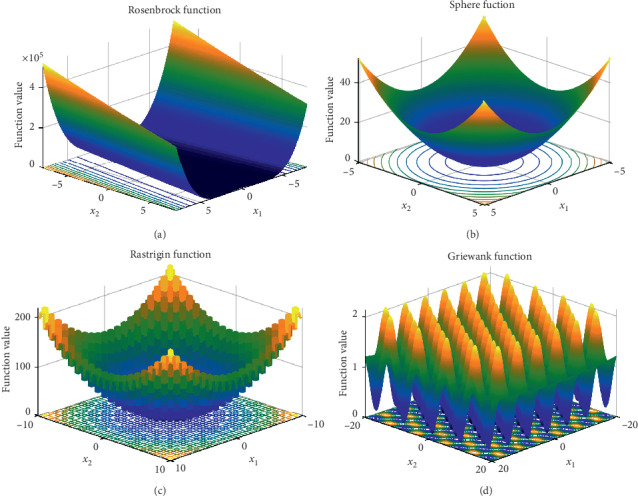
3D figure of the test functions.

The SCBFO algorithm was investigated with 10 test functions provided by the CEC 2015 benchmark test set [24], shown in Table4 and Figure 6. Because these 10 test functions are commonly used key functions selected by related literature [14–19], including the new algorithms (CEBFO and MBFO) used in this experiment, it is convincing to process the experiment with these test functions in the validation of the SCBFO algorithm. The first five test functions in Table 4 are unimodal functions, and the others are multimodal functions. However, for each of the ten functions, there is only one unique global optimal solution in the optimization space.

Under the same test conditions, SCBFO was tested and compared with the classical BFO algorithm and the classical PSO algorithm.

At the same time, two newly improved bacteria foraging algorithms in recent years, CEBFO (2017) [14] and MBFO (2015) [19], were also taken into account and compared with the SCFBO algorithm proposed in this paper.

To guarantee the fairness and credibility of algorithms in comparison, some initializations were determined preliminarily. The initial parameters of the classical BFO, the CEBFO, the MBFO, and the SCBFO algorithm maintained the same. Besides, the iteration number of the classical PSO algorithm was defined as *T*=*N*_*c*_*∗N*_re_*∗N*_ed_ to make it comparable with other algorithms. The essential parameter settings of five algorithms are shown in Table 5.

According to the calculation flow in Section 3.3, because the solution spaces vary among the test functions, the initial step size of bacterial swimming *C* is different according to the order of the solution space, as shown in Table 5.

Under the test conditions of 5, 10, 30, and 50 dimensions (*D* = 5, 10, 30, and 50), the five algorithms were calculated 100 times, respectively. Then, the computational results were collected and analyzed.

### 4.2. Results and Comparison

After the computation, the convergence trend and typical data such as the best value, the worst value, the mean value, and the variance can be achieved from the computed search results, which will be used in the following analysis on the performance of the SCBFO algorithm.

Since the trends of the computed results in different dimensions are similar for a certain test function and a higher dimension makes the optimization more challenging, the highest dimension *D* = 50 is chosen to illustrate the feasibility and the advantages of the SCBFO. The search results with dimension *D* = 50 are listed in Table 6.

For better understanding, the comparison among the SCBFO algorithm and other algorithms is processed in the optimization results, the convergence trend, and the performance stability, respectively.

#### 4.2.1. Comparison of Optimization Results

**Figure 7:**
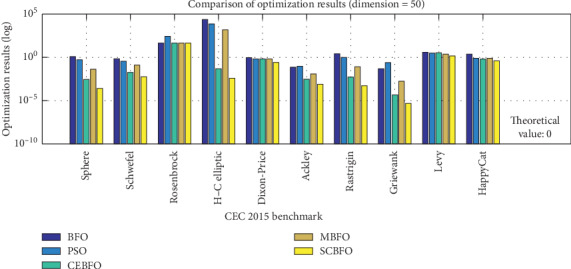
The comparison of the results.

The optimization results are listed in Table 6. According to the mean value of each test in Table 6, the comparison in Figure 7 can be formed, which is logarithmically arranged to achieve a unified contrast.

From Table 4, we can see the theoretical value of the optimal solutions in the test set is 0. As shown clearly in Table 6 and Figure 7, we find that, for each of the 10 test functions, the optimization result of the SCBFO algorithm remains the smallest, which means the SCBFO algorithm gets the optimal solution closest to the theoretical value. Even compared with other improved algorithms including CEBFO and MBFO, the SCBFO algorithm has higher accuracy in the optimization results.

Therefore, the SCBFO algorithm reduces the risk of local convergence to a large extent and improves the accuracy when searching for the global optimum, which proves the SCBFO algorithm is effective in the optimization.

#### 4.2.2. Comparison of Convergence Trends

**Figure 8:**
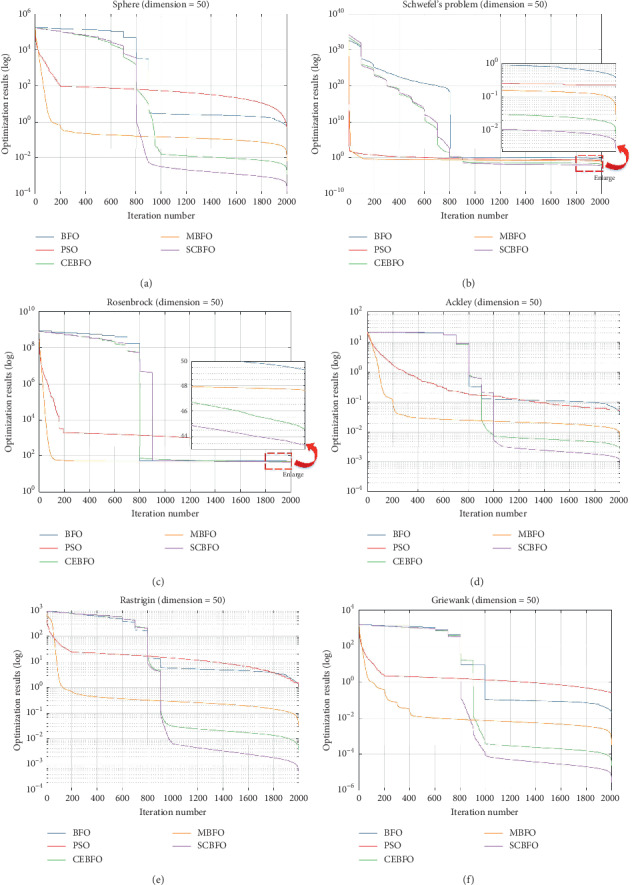
The comparison of convergence trends.

To evaluate the performances of an algorithm, the exploration ability in the early stage and the exploitation ability in the later stage are also the key standards. The mean values in different processes during the iteration are calculated according to the 100 times of computation with five kinds of algorithms. The convergence trends are plotted in Figure 8 and also logarithmically presented for clearer illustration.

As shown in Figure 8, in the exploration stage *T* < 40% (iteration number *t* < 800), the convergence of the SCBFO algorithm is not as fast as the classical BFO and the PSO algorithm because of a large-scale exploration search. However, the SCBFO algorithm shows the continuous convergence trend similar to the CEBFO and the MBFO algorithm, which means the SCBFO algorithm can keep a sufficient exploration stage for the large-scale exploration search.

Then, in the exploitation stage *T* > 40% (iteration number *t* > 800), especially 90% < *T* < 100% (iteration number 1,800 < *t* < 2,000), the SCBFO algorithm shows high convergence speed and excellent optimization results. At this stage, the convergence speed of the classical BFO and the PSO algorithm is reduced or even stopped when the algorithm reaches the optimal solution of 10^−1^ order. This proves that their exploitation activity in this stage is so weak that they are more likely to get into the local optimum. On the contrary, compared with other algorithms, SCBFO in this stage keeps a considerable declining speed until the end of the iterative process, which accelerates the process of convergence.

Therefore, the SCBFO algorithm is proved to effectively balance the exploration and the exploitation, which means the SCBFO algorithm can reduce the risk of falling into local optimum and make the convergence to optimal results more reliable.

#### 4.2.3. Comparison of Performance Stability

**Figure 9:**
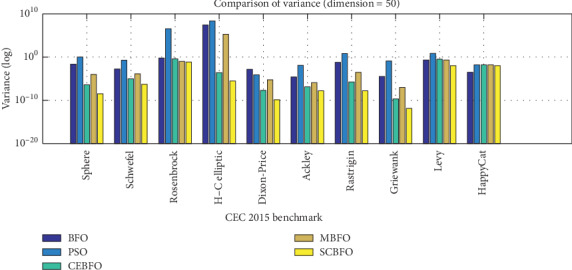
Variance comparison of 100 computations.

**Figure 10:**
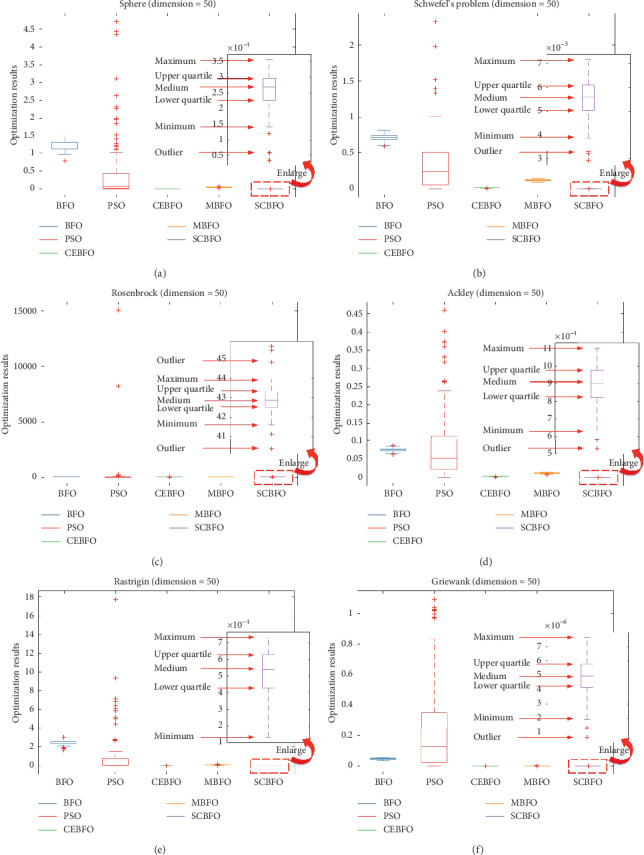
Box graph of the best, worst, and variance.

As a swarm intelligence algorithm with wide application prospects, the SCBFO algorithm also needs to take the performance stability as one of the key standards to evaluate its performances. This section focuses on the variance, the best value, and the worst value according to the 100 times of computation with each algorithm in the highest dimension *D* = 50, as shown in Table 7. And Figure 9 plots all the variances logarithmically. Meanwhile, all results with the dimension *D* = 50 are rearranged as discrete data statistics used for the box graph in Figure 10. The box graph in Figure 10 can clearly show the stability differences of five algorithms under the current dimension in each test function.

As shown in Figures 9 and 10 and Table 7, the SCBFO algorithm shows the best variance. It can be seen that the results of the classical BFO algorithm and PSO algorithm fluctuate considerably. The classical BFO and PSO algorithms have a lot of outliers and a wider quartile range, which is in accord with the regularity delivered by Table 7 and indicates their unstable performances. On the contrary, referring to the variances in the box graph, the computed results of the SCBFO algorithm show the smallest medium value, the most concentrated quartile range, and the least outlier value.

Therefore, it is proved that the SCBFO algorithm shows the strongest stability of search performance among the five algorithms, which means the SCBFO algorithm proposed in this research will be more suitable to complex real-world applications of optimization.

## 5. Conclusions

To overcome the fixed step size and the weak correlation among bacteria of the classical BFO algorithm, the SCBFO algorithm was proposed in this paper. The self-adaptive chemotaxis strategy was designed by proposing the self-adaptive swimming method based on bacterial search state features and improving the chemotaxis flipping based on information exchange strategy.

The SCBFO algorithm was tested and verified by the CEC 2015 benchmark test set and compared with the classical BFO, the classical PSO, and two improved bacteria foraging algorithms in recent years: the CEBFO and the MBFO algorithm. The validation results proved the SCBFO algorithm effective and accurate in obtaining the optimal solution. Meanwhile, the stronger exploitation ability in the later stage and the more stable search performance of the SCBFO algorithm were also illustrated.

To sum up, the SCBFO algorithm does well in balancing the exploration and the exploitation and reducing the risk of local convergence, which means it can overcome the aforementioned two drawbacks of the classical BFO. Meanwhile, the SCBFO algorithm presents excellent search performance stability. Therefore, the SCBFO provides a novel and efficient theory to deal with complex optimization tasks.

## Figures and Tables

**Table 1:** The fuzzy sets.

S	Small
M	Medium
B	Large
NEB	Negative extreme large
NB	Negative large
NS	Negative small
ZE	Zero
PS	Positive small
PB	Positive large
EB	Extreme large

**Table 2:** The fuzzy rules of d*n* (*C*_Multi_).

T	d*J*(*t*)/div(*t*)	EB	B	M	S	ZE

S		ZE (ZE)	PS (ZE)	PB (PS)	PB (PS)	PB (PS)

M	NB	PS (PS)	PB (PS)	PB (PS)	PB (PS)	PB (PS)
	NS	ZE (PS)	PS (PS)	PS (PS)	PB (PS)	PB (PS)
	ZE	ZE (ZE)	ZE (ZE)	PS (PS)	PS (PS)	PB (PS)
	PS	NB (ZE)	NS (ZE)	ZE (ZE)	PS (PS)	PB (PS)
	PB	NB (NB)	NS (NS)	NS (NS)	ZE (ZE)	PB (PS)

B	NB			NS (ZE)	ZE (PS)	PB (PS)
	NS			NS (NS)	ZE (ZE)	PS (ZE)
	ZE			NS (NS)	NS (NS)	ZE (ZE)
	PS			NB (NB)	NS (NB)	ZE (NB)
	PB			NB (NEB)	NB (NEB)	NB (NEB)

**Table 3:** Essential parameter settings.

Parameters	Description
*D*	Dimension of the optimization space
*S*	Total number of the bacteria
*C*	Initial step size of bacterial swimming
*P *_ed_	Probability of bacteria elimination and dispersal
*N *_*c*_	Maximum number of chemotaxis
*N *_*S*_	Maximum number of bacterial swimming
*N *_re_	Maximum number of reproduction
*N *_ed_	Maximum number of elimination and dispersal
*P *_*i*_ (*j*,*k*,*l*)	The bacterial initial position
ω	Inertia factor
*C *_1_ and *C*_2_	Acceleration constants
*R *_1_ and *R*_2_	Random values between 0 and 1

**Table 4:** The test functions of CEC 2015.

Function	Formulation	Limits	Minimum
Type: unimodal			
Sphere	*f*_1_(*x*)=∑_*i*=1_^*D*^*x*_*i*_^2^	*x*_*i*_ ∈ [−100,100]	$f_{1}\left(\overset{⟶}{0}\right)=0$
Schwefel's problem 2.22	*f*_2_(*x*)=∑_*i*=1_^*D*^|*x*_*i*_|+∏_*i*=1_^*D*^|*x*_*i*_|	*x*_*i*_ ∈ [−10,10]	$f_{2}\left(\overset{⟶}{0}\right)=0$
Rosenbrock	*f*_3_(*x*)=∑_*i*=1_^*P*−1^[100(*x*_*i*+1_ − *x*_*i*_)^2^+(1 − *x*_*i*_)^2^]	*x*_*i*_ ∈ [−30,30]	$f_{3}\left(\overset{⟶}{0}\right)=0$
High conditioned elliptic	*f*_4_(*x*)=∑_*i*=1_^*D*^(10^6^)^((*i* − 1)/(*D* − 1))^*x*_*i*_^2^	*x*_*i*_ ∈ [−100,100]	$f_{4}\left(\overset{⟶}{0}\right)=0$
Dixon-Price	*f*_5_(*x*)=(*x*_1_ − 1)^2^+∑_*i*=2_^*D*^*i*(2*x*_*i*_^2^ − *x*_*i*−1_)^2^	*x*_*i*_ ∈ [−10,10]	$f_{5}\left(\overset{⟶}{x_{i}}\right)=0$

Type: multimodal			
Ackley	$f_{6}\left(x\right)=-20\exp\left(-0.2\sqrt{\left(1/P\right)\sum_{i=1}^{P}x_{i}^{2}}\right)-\exp\left(\left(1/P\right)\sum_{i=1}^{P}\cos\left(2\pi x_{i}\right)\right)+20+e$	*x*_*i*_ ∈ [−30,30]	$f_{6}\left(\overset{⟶}{0}\right)=0$
Rastrigin	*f*_7_(*x*)=∑_*i*=1_^*P*^(*x*_*i*_^2^ − 10cos(2*πx*_*i*_)+10)	*x*_*i*_ ∈ [−5.12, 5.12]	$f_{7}\left(\overset{⟶}{0}\right)=0$
Griewank	$f_{8}\left(x\right)=\left(1/4000\right)\left(\sum_{i=1}^{P}x_{i}^{2}\right)-\left[\prod_{i=1}^{P}\cos\left(x_{i}|/|\sqrt{i}\right)\right]+1$	*x*_*i*_ ∈ [−600,600]	$f_{8}\left(\overset{⟶}{0}\right)=0$
Levy	*f*_9_(*x*)=sin^2^(*πω*_1_)+∑_*i*=1_^*D*−1^(*ω*_*i*_ − 1)^2^[1+10sin^2^(*πω*_*i*_+1)]+(*ω*_*D*_ − 1)^2^[1+sin^2^(2*πω*_*D*_)]; *ω*_*i*_=1+((*x*_*i*_ − 1)/4)	*x*_*i*_ ∈ [−10,10]	$f_{9}\left(\overset{⟶}{1}\right)=0$
HappyCat	*f*_10_(*x*)=|∑_*i*=1_^*D*^*x*_*i*_^2^−*D*|^1/4^+((0.5∑_*i*=1_^*D*^*x*_*i*_^2^+∑_*i*=1_^*D*^*x*_*i*_)/*D*)+0.5	*x*_*i*_ ∈ [−5,5]	$f_{10}\left(\overset{⟶}{-1}\right)=0$

**Table 5:** The initial parameters of five algorithms.

Algorithm	Essential parameter settings
SCBFO/BFO/CEBFO/MBFO/PSO	*S* = 50 (bacteria and particles); *C* = 0.01; *P*_ed_ = 0.25; *N*_*c*_ = 100; *N*_*S*_ = 4; *N*_re_ = 5; *N*_ed_ = 4
PSO	*T*=*N*_*c*_*∗N*_re_*∗N*_ed_=2000
SCBFO/CEBFO	*ω* = 0.9; *C*_*1*_=*C*_*2*_=2

**Table 6:** All optimization results (dimension = 50) (italics is the worst; bold is the best).

Function	BFO	PSO	CEBFO	MBFO	SCBFO
Sphere	*1.2296e + 00*	5.1629*e* − 01	2.9785*e* − 03	4.3782*e* − 02	**2.5640e − 04**
Schwefel	*7.1727e − 01*	3.6523*e* − 01	1.8411*e* − 02	1.2251*e* − 01	**5.4840e − 03**
Rosenbrock	4.8971*e* + 01	*2.6187e + 02*	4.3895*e* + 01	4.7577*e* + 01	**4.2921e + 01**
H-C elliptic	*2.3254e + 04*	7.5619*e* + 03	4.5441*e* − 02	1.4744*e* + 03	**3.7319e − 03**
Dixon-Price	*9.7442e − 01*	6.6671*e* − 01	6.6706*e* − 01	6.8092*e* − 01	**2.5206e − 01**
Ackley	7.5556*e* − 02	*9.5134e − 02*	3.0657*e* − 03	1.2194*e* − 02	**8.9346e − 04**
Rastrigin	*2.4519e + 00*	1.0396*e* + 00	5.6716*e* − 03	8.4771*e* − 02	**5.2069e − 04**
Griewank	4.9338*e* − 02	*2.6284e − 01*	5.1255*e* − 05	1.7769*e* − 03	**4.8724e − 06**
Levy	*3.9906e + 00*	3.2803*e* + 00	3.2916*e* + 00	2.4980*e* + 00	**1.6172e + 00**
HappyCat	*2.4611e + 00*	8.2289*e* − 01	6.1940*e* − 01	8.0924*e* − 01	**4.1961e − 01**

**Table 7:** All best results, worst results, and variance (dimension = 50) (italics is the worst; bold is the best).

Function		BFO	PSO	CEBFO	MBFO	SCBFO
Sphere	Var	1.8168*e* − 02	*9.6163e − 01*	4.0667*e* − 07	7.9810*e* − 05	**3.1818e − 09**
	Max	1.5580*e* + 00	*4.7097e + 00*	4.3795*e* − 03	6.6067*e* − 02	**3.5465e − 04**
	Min	*7.7258e − 01*	3.4255*e* − 05	1.3526*e* − 03	1.9721*e* − 02	**1.0572e − 07**

Schwefel	Var	1.6688*e* − 03	*1.7598e − 01*	6.9786*e* − 06	1.2129*e* − 04	**6.8157e − 07**
	Max	8.2152*e* − 01	*2.3245e + 00*	2.3840*e* − 02	1.4782*e* − 01	**7.1639e − 03**
	Min	*5.9840e − 01*	3.1871*e* − 03	9.3401*e* − 03	9.7139*e* − 02	**2.9258e − 03**

Rosenbrock	Var	5.8421*e* − 01	*2.9041e + 06*	3.0092*e* − 01	9.1829*e* − 02	**6.5959e − 02**
	Max	4.9550*e* + 01	*1.5035e + 04*	4.4896*e* + 01	4.8183*e* + 01	**4.5619e + 01**
	Min	*4.8350e + 01*	4.9573*e* − 04	4.2292*e* + 01	4.6823*e* + 01	**4.0341e + 01**

H-C elliptic	Var	1.7698*e* + 07	*1.4887e + 08*	2.4810*e* − 04	1.4062*e* + 05	**2.8406e − 06**
	Max	3.2909*e* + 04	*6.4013e + 04*	9.1198*e* − 02	2.6512*e* + 03	**8.9012e − 03**
	Min	*1.3529e + 04*	9.5535*e* − 02	1.4314*e* − 02	7.1351*e* + 02	**9.2632e − 04**

Dixon-Price	Var	*1.3027e − 03*	7.1421*e* − 05	1.4317*e* − 08	4.5475*e* − 06	**1.7083e − 10**
	Max	*1.0468e + 00*	6.6674*e* − 01	6.6744*e* − 01	6.8667*e* − 01	**2.9926e − 01**
	Min	*8.6503e − 01*	6.6669*e* − 01	6.6683*e* − 01	6.7661*e* − 01	**2.0882e − 01**

Ackley	Var	2.0700*e* − 05	*1.1686e − 02*	1.2173*e* − 07	1.2746*e* − 06	**1.3719e − 08**
	Max	8.7222*e* − 02	*4.5927e − 01*	3.7252*e* − 03	1.4666*e* − 02	**1.1020e − 03**
	Min	*6.2410e − 02*	3.3865*e* − 04	1.6901*e* − 03	7.7651*e* − 03	**5.2645e − 04**

Rastrigin	Var	5.9407*e* − 02	*6.2404e + 00*	1.9758*e* − 06	2.5533*e* − 04	**1.8916e − 08**
	Max	2.9923*e* + 00	*1.7757e + 01*	8.3655*e* − 03	1.5550*e* − 01	**7.3435e − 04**
	Min	*1.6563e + 00*	1.2970*e* − 04	1.4597*e* − 03	4.1051*e* − 02	**1.0689e − 04**

Griewank	Var	2.5248*e* − 05	*1.0517e − 01*	1.9018*e* − 10	1.2555*e* − 07	**1.4983e − 12**
	Max	5.8761*e* − 02	*1.0910e + 00*	8.2053*e* − 05	2.9404*e* − 03	**7.6257e − 06**
	Min	*3.7490e − 02*	3.2241*e* − 05	1.4332*e* − 05	9.6940*e* − 04	**6.6235e − 07**

Levy	Var	1.8155*e* − 01	*5.1672e + 00*	2.2766*e* − 01	1.5822*e* − 01	**9.1610e − 03**
	Max	4.3065*e* + 00	*2.1043e + 01*	4.3936*e* + 00	4.3933*e* + 00	**3.4090e + 00**
	Min	*3.7667e + 00*	2.0733*e* + 00	2.0732*e* + 00	1.7975*e* + 00	**7.0299e − 01**

HappyCat	Var	**2.5977e − 04**	*1.3614e − 02*	1.3554*e* − 02	1.3337*e* − 02	8.3045*e − *03
	Max	*2.4948e + 00*	1.1517*e* + 00	1.1308*e* + 00	9.6111*e* − 01	**7.5124e − 01**
	Min	*2.4106e + 00*	4.9291*e* − 01	5.1663*e* − 01	4.3305*e* − 01	**1.6869e − 01**

## Data Availability

The related benchmark problems used to support the findings of this study can be found in this article or the website (https://github.com/P-N-Suganthan).
